# Supplementary material for: Spatio-Temporal Variations in Pollen Limitation and Floral Traits of an Alpine Lousewort (Pedicularis rhinanthoides) in Relation to Pollinator Availability
Source: Plants (Basel). 2022 Dec 23;12(1):78. doi: 10.3390/plants12010078 (PMC9824556; doi:10.3390/plants12010078)
Supplement: Supplementary file 1 [file plants-12-00078-s001.zip › plants-2021177-supplementary.pdf]

Supplementary Table S1. Three-way ANOVA of the effect of different pollination treatments, flowering stages, and populations site on seed production per capsule in pollen supplemental experiments.

Variable	Seed production per capsule			
Effect	*df*	*MS*	*F*	*P*
Treatment	1	524.348	47.858	< 0.001
Stage	2	61.661	5.628	0.004
Population	2	50.925	4.648	0.01
Treatment × stage	2	3.76	0.343	0.71
Treatment × population	2	10.236	0.934	0.394
Stage × population	4	2.456	0.224	0.925
Treatment × stage × population	4	0.28	0.026	0.999
Error	475	10.956		
Total	493			
